# Expression of RNA interference triggers from an oncolytic herpes simplex virus results in specific silencing in tumour cells *in vitro *and tumours *in vivo*

**DOI:** 10.1186/1471-2407-10-486

**Published:** 2010-09-13

**Authors:** Anna-Maria Anesti, Guy R Simpson, Toby Price, Hardev S Pandha, Robert S Coffin

**Affiliations:** 1 Research Group, Biovex Inc, Commerce Way, Woburn, MA1801, USA; 2 Oncology Group, Postgraduate Medical School, University of Surrey, Surrey, GU2 5XH, UK

## Abstract

**Background:**

Delivery of small interfering RNA (siRNA) to tumours remains a major obstacle for the development of RNA interference (RNAi)-based therapeutics. Following the promising pre-clinical and clinical results with the oncolytic herpes simplex virus (HSV) OncoVEX^GM-CSF^, we aimed to express RNAi triggers from oncolytic HSV, which although has the potential to improve treatment by silencing tumour-related genes, was not considered possible due to the highly oncolytic properties of HSV.

**Methods:**

To evaluate RNAi-mediated silencing from an oncolytic HSV backbone, we developed novel replicating HSV vectors expressing short-hairpin RNA (shRNA) or artificial microRNA (miRNA) against the reporter genes green fluorescent protein (eGFP) and β-galactosidase (lacZ). These vectors were tested in non-tumour cell lines *in vitro *and tumour cells that are moderately susceptible to HSV infection both *in vitro *and in mice xenografts *in vivo*. Silencing was assessed at the protein level by fluorescent microscopy, x-gal staining, enzyme activity assay, and western blotting.

**Results:**

Our results demonstrate that it is possible to express shRNA and artificial miRNA from an oncolytic HSV backbone, which had not been previously investigated. Furthermore, oncolytic HSV-mediated delivery of RNAi triggers resulted in effective and specific silencing of targeted genes in tumour cells *in vitro *and tumours *in vivo*, with the viruses expressing artificial miRNA being comprehensibly more effective.

**Conclusions:**

This preliminary data provide the first demonstration of oncolytic HSV-mediated expression of shRNA or artificial miRNA and silencing of targeted genes in tumour cells *in vitro *and *in vivo*. The vectors developed in this study are being adapted to silence tumour-related genes in an ongoing study that aims to improve the effectiveness of oncolytic HSV treatment in tumours that are moderately susceptible to HSV infection and thus, potentially improve response rates seen in human clinical trials.

## Background

RNA interference (RNAi) has emerged not only as a powerful tool in functional genomics and the validation of novel gene targets in drug discovery, but also as a potential therapeutic strategy for diverse diseases, including cancer [1]. RNAi is a post-transcriptional gene silencing mechanism mediated by small double-stranded RNA (dsRNA) molecules, including small interfering RNAs (siRNAs) and microRNAs (miRNAs) [2-6]. In mammalian cells, RNAi can be induced by synthetic siRNAs introduced directly into the cell or by plasmid and viral vectors that express short-hairpin RNA (shRNA) or artificial miRNAs, which have been termed the new generation RNAi triggers [7-9].

Although a variety of strategies, such as chemical modifications, liposomes, nanoparticles, and antibodies or cell-surface receptors, have been employed to increase siRNA stability and delivery to specific cell types [10], *in vivo *delivery of siRNAs remains a major obstacle for the development of RNAi-based cancer therapeutics. As a more efficient alternative, replication-defective viral vectors have been developed to silence genes in tumours. A retroviral vector has demonstrated silencing of HER-2/neu [11]. Lentiviral vectors have been employed to silence a number of tumour-associated genes, including Tiam1, resulting in suppression of cancer cell growth *in vitro *and *in vivo *[12]. Similar inhibition of tumour cell growth has been achieved by adenoviruses expressing shRNA against ASH1, p28GANK, Skp-2 and Hec1 [13-15]. Furthermore, herpes simplex virus (HSV) amplicon vectors have been shown to silence genes in tumour cells both *in vitro *and *in vivo *[16,17].

Replicating viruses engineered to exhibit selective tumour cytotoxicity have a significant advantage over non-replicating viruses. A number of oncolytic viruses are currently under clinical development for cancer therapy. Thus, there is considerable interest in expressing shRNA from these viruses to improve their tumour killing properties. Oncolytic adenovirus expressing shRNA against the firefly luciferase transgene achieved 30% silencing in a number of tumour cell lines [18]. More recently, replication-competent adenoviruses expressing shRNA against vascular endothelial growth factor (VEGF) and Interleukin-8 (IL-8) have been shown to affect angiogenesis and inhibit tumour growth [19,20].

OncoVEX is a second-generation oncolytic HSV-1 with deletion in ICP34.5 to provide tumour selective replication [21,22] and deletion of ICP47 resulting in the expression of the US11 gene as an immediate-early (IE) rather than a late (L) gene to further increase tumour replication [23]. Infection of cells with wild type HSV reduces antigen expression on the cell surface through the expression of ICP47, which inhibits the transporters associated with antigen presentation (TAP) [24,25]. Therefore, deletion of ICP47 would be expected to increase the anti-tumour immune response in the presence of HSV [26,27]. This oncolytic HSV-1 backbone has demonstrated improved tumour shrinkage properties compared to previously developed oncolytic viruses and has been used to successfully express a range of therapeutic genes in pre-clinical testing, including granulocyte macrophage colony-stimulating factor (GM-CSF), retroviral glycoprotein, pro-drug activating system, and Tumor Necrosis Factor-Alpha (TNFα) [28-30]. OncoVEX^GM-CSF ^has been further tested in Phase I and Phase II clinical trials by direct injection into a number of tumour types with promising results [31], and has entered Phase III clinical studies in melanoma. While enhanced activity has been observed when therapeutic genes have been inserted into oncolytic HSV-1 genomes, expression of shRNA had not been previously investigated.

Following the promising pre-clinical and clinical results with OncoVEX, the current study aimed to identify whether it was possible to express shRNA and artificial miRNA from an oncolytic HSV backbone. To this end, we developed viruses expressing shRNA or pre-miRNA against β-galactosidase (lacZ) or enhanced green fluorescent protein (eGFP) and evaluated the potential of these vectors both *in vitro *and *in vivo*. We demonstrate, for the first time, that shRNA and artificial miRNA can be expressed from an oncolytic HSV virus and results in effective silencing of reporter genes *in vitro *in non-tumour cells that are highly susceptible and tumour cells that are moderately susceptible to HSV infection and *in vivo *in tumours that are not cured by oncolytic virus treatment alone. Ultimately, by down regulating the levels of proteins secreted by tumour cells, which aid their growth or promote local immune suppression, we may further improve tumour cell killing by oncolytic HSV in tumours that are moderately susceptible to HSV oncolysis. In the clinical situation, this is expected to increase the response rates seen. For example, in melanoma, response rates were approximately 30% in a phase II clinical trial and therefore, increasing this rate of response using RNAi, or other approaches, would be clearly clinically beneficial.

## Methods

### Cell lines

All cell lines were obtained from either ATCC or ECACC or produced by the authors and were grown at 37°C in recommended media under a humidified atmosphere of 5% CO_2_. The following cell lines were used: BHK-21 (clone 13) (85011433), BHK-21 LacZ [32] and 9L *lacZ *(ATCC-CRL-2200) [33].

### Construction and characterisation of viral vectors

Production of the oncolytic vector OncoVEX is described by ref. 26. The cassettes expressing shRNA or artificial miRNA against *lacZ *or enhanced green fluorescent protein (*eGFP*) are described by ref. 32. They express shRNA and pre-miRNA sequences from the U6 polymerase III and CMV polymerase II promoters, respectively. The pR19 cassette is flanked by HSV-1 LAT sequences.

### B-galactosidase activity assay

B-galactosidase enzyme activity was assessed using high sensitivity β-galactosidase assay kit (Stratagene). BHK-LacZ cells were plated at 1.5×10^5 ^cells per well of 24-well tray and incubated at 37°C overnight. Cells were infected with Onc miR-LacZ, Onc miR-neg, Onc U6shLacZ and Onc U6shGFP at multiplicity of infection (MOI) of 1, 0.1 and 0.01 and incubated at 37°C for 24 hours. Cells were freeze-thawed in lysis buffer and spun at 12,0000 g for 5 mins to remove cell debris. 20 ul of each cell lysate was added to the wells of a 96 well micotiter ELISA dish (*IWAKI *ELISA). 130 ul of chlorophenol red-β-D-galactopyranoside substrate was added and the samples were incubated at 37°C until the substrate colour changed from yellow to brown. The reactions were terminated by 80 ul of stop solution. The plate was read using an OPSYS MR plate reader (DYNEX technologies) at OD ^570-595^.

### Western blot analysis

B-galactosidase protein levels were assessed by standard Western blot techniques, performed on 20 ug of protein per sample and using primary antibodies: β-galactosidase (Abcam; ab616), a-tubulin (Abcam; ab4074) and secondary antibody: goat polyclonal to rabbit IgG-H&L-HRP (Abcam).

### *In vivo *xenograft tumour models

All experimental procedures were performed with the authority of the Home Office, following UK guidelines and in accordance with best animal practice. Rat 9L/LacZ cells were implanted (1×10^7 ^cells) subcutaneously in the right flank of BALB/c nude mice (Harlan laboratories). Tumours were allowed to develop for 15 days to an average diameter of approximately 0.4-0.6 cm and then injected with 10^8 ^pfu (100 μl) of either Onc miR-LacZ or Onc miR-neg, and further incubated for 72 hrs. B-galactosidase protein levels were either assessed by standard Western blot techniques or high sensitivity β-galactosidase assay kit (Stratagene).

### MTS cell viability assay and viral growth curves

BHK-LacZ cells, were seeded in 96-well plates at a density of 2.5×10^4 ^cells per well and incubated at 37°C O/N. Cells were infected with Onc miR-LacZ, Onc U6shLacZ or Oncovex backbone at a multiplicity of infection (MOI) of 1, 0.1 and 0.01, and incubated at 37°C for 24, 48 and 72 hours. At each time point the supernatants were removed (stored at -80°C) and the viral titre was determined using a standard viral plaque assay. Cell viability was determined using the CellTiter 96 AQueous One Solution Cell Proliferation Assay reagent 3-(4, 5-dimethylthiazol-2-yl) -5- (3-carboxymethoxyphenyl) -2- (4-sulfophenyl) -2H- tetrazolium) (MTS) according to the manufacturer's instructions (Promega). Briefly, 20 μL of MTS reagent were added to each well together with 180 ul 2% FCS, RMPI, followed by incubation at 37°C for 15 min - 4 hours. Absorbance was measured at 495 nm. Survival was calculated as a percentage of the measurements taken in untreated cells.

## Results

### Development of oncolytic HSV RNAi viruses

The polymerase III U6 promoter has been most commonly used to express shRNA from plasmid and viral vector systems. However, shRNA can be efficiently expressed from the CMV polymerase II promoter when embedded into endogenous miRNA sequences and these artificial miRNAs have been shown to induce specific degradation of target mRNAs similar to transfected siRNAs [34-36]. The microRNA-like expression system used in this study is based on miR-155 and takes advantage of the flexibility and variety of polymerase II promoters, which unlike polymerase III promoters, can be inducible and tissue-specific [37]. A reporter gene encoding emerald GFP is co-expressed from this system and allows labelling of transduced cells to aid the monitoring of silencing efficiency.

**Figure 1:**
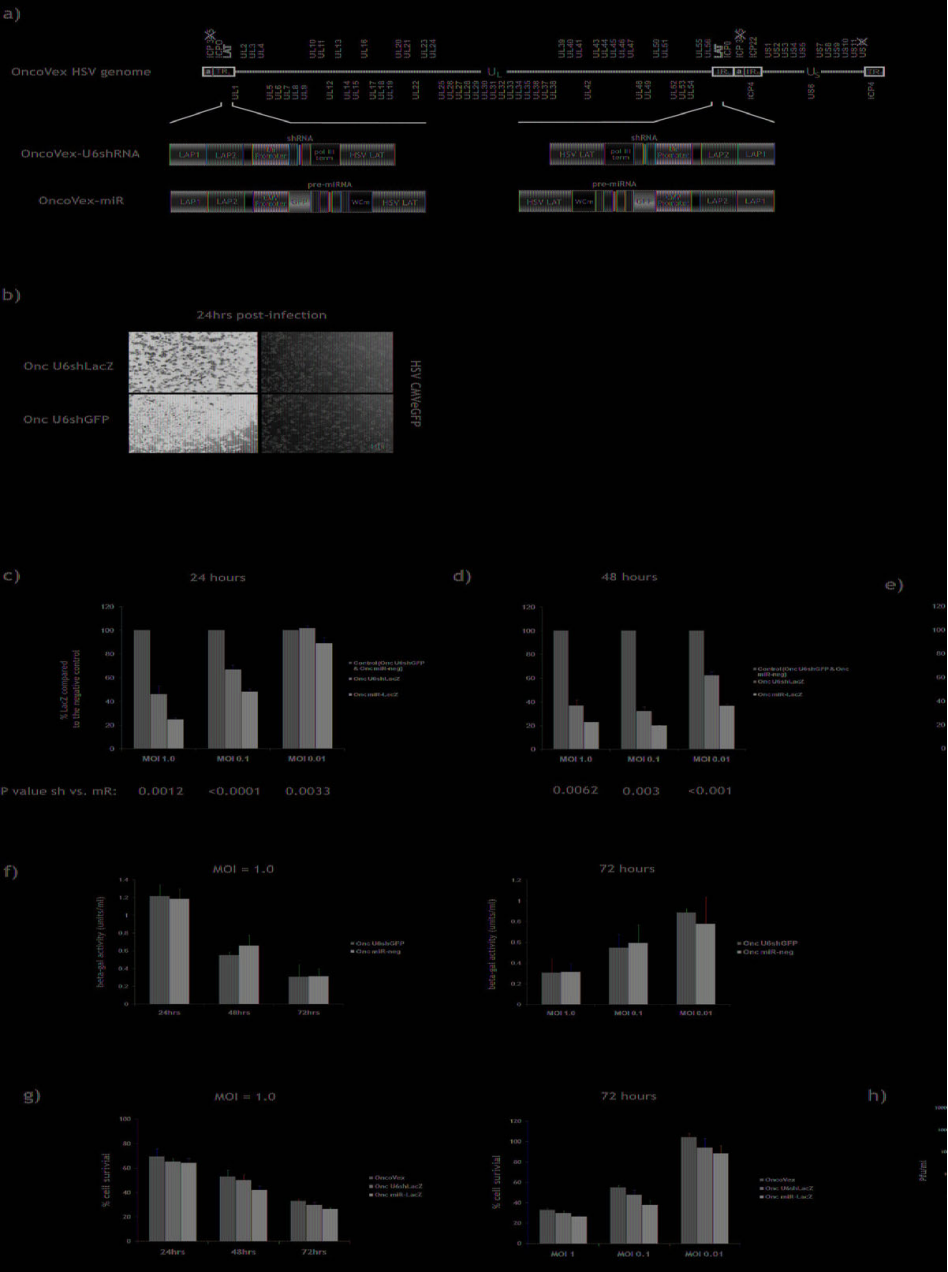
**Construction and characterisation of oncolytic HSV viruses expressing shRNA**. (**a**) Schematic representation of the oncolytic HSV genome and RNAi expression cassettes used in this study. Generation of OncoVEX viruses is described by Liu *et al*., 2003 and generation of replication-defective HSV-1 vectors is described by Anesti *et al.*, 2008. (**b**) BHK cells were dually infected with a non-replicating HSV CMVeGFP virus and either Onc U6shGFP or Onc U6shLacZ, at an MOI of 1.0, and incubated for 24 hrs. The cells were digitally photographed using an UV-inverted microscope (*Nikon *Eclipse TE200) and *Lucia *Imaging (MV-1500 version 4.6). (**c,d,e**) BHK-LacZ cells ^29 ^were infected at various MOIs (1, 0.1, and 0.01) with oncolytic HSV viruses expressing shRNA (Onc U6shLacZ) or pre-miRNA (Onc miR-LacZ) against lacZ, or the negative control viruses Onc U6shGFP and Onc miR-neg. Infected cells were harvested at 24, 48 and 72 hrs and β-galactosidase protein levels were assessed by high sensitivity β-galactosidase assay (Stratagene). B-galactosidase levels (%LacZ) were expressed as a percentage of the expression measured in the cells infected with the negative controls (Onc U6shGFP for Onc U6shLacZ, and Onc miR-neg for Onc miR-Lac. (**f**) B-galactosidase activity (units/ml) in BHK-LacZ cells infected with Onc U6shGFP and Onc miR-neg control viruses at an MOI 1.0, and incubated for 24 hrs, 48 hrs and 73 hrs, or at an MOI 1.0, 0.1 and 0.01, and incubated at 72 hrs. BHK-LacZ cells constitutively express lacZ and thus, β-galactosidase levels are a good indication of cell survival. All P values were obtained using unpaired student t test. **(g) **BHK-LacZ cells were infected with OncoVEX, Onc U6shLacZ or Onc miR-LacZ at an MOI of 1.0, 0.1 and 0.01. MTS cell viability assay was performed at 24, 48 and 72 hours post-infection. Cell death increases with time and MOI of virus. Expression of shLacZ or miR-LacZ does not significantly affect cytotoxicity caused by the oncolytic virus. **(h) **BHK-LacZ cells were infected with OncoVEX, Onc U6shLacZ or Onc miR-LacZ. Growth curves were prepared by counting plaque forming units at 24, 48 and 72 hours post-infection. Expression of shLacZ or miR-LacZ does not significantly influence the growth of the oncolytic virus.

RNAi viruses based on the OncoVEX oncolytic HSV backbone [28-30] were constructed by inserting cassettes expressing shRNA or miRNA against the *gfp *or *lacZ *reporter genes into the latency associated transcript (LAT) region of the HSV genome (Figure 1a). Expression of shRNA and pre-miRNA was driven by the U6 polymerase III and CMV polymerase II promoters, respectively. These RNAi cassettes have been described previously and showed highly effective and specific silencing of reporter and endogenous gene expression in neurons, when inserted into replication-defective HSV-1 vectors [32].

### Oncolytic HSV can mediate expression of shRNA

To identify whether shRNA could be expressed from an oncolytic HSV, BHK cells were co-infected with a non-replicating HSV vector expressing eGFP (HSV CMVeGFP) and either the oncolytic virus Onc U6shGFP expressing shRNA against GFP or Onc U6shLacZ expressing shRNA against β-galactosidase, at an MOI 1.0. BHK cells are highly susceptible to HSV infection and at 24 hrs over 90% of cells are GFP positive. Furthermore, whilst the majority of cells are infected with replicating HSV (cells with rounded morphology), only very few dead cells were detected in the media and the cultures appeared healthy. At 24 hrs post-infection, cells infected with Onc U6shGFP showed a significant reduction in GFP expression levels compared to the negative control (Figure 1b). Onc U6shLacZ failed to reduce GFP expression indicating that the silencing observed is not mediated by a non-specific effect caused by the oncolytic HSV backbone. We can therefore conclude that it is possible to express active shRNA from an oncolytic HSV virus.

### Evaluation of oncolytic HSV-mediated expression of shRNA and miRNA

To optimise silencing from oncolytic HSV, we compared viruses expressing shRNA or artificial miRNA against β-galactosidase from the U6 (Onc U6shLacZ) and CMV (Onc miR-LacZ) promoters, respectively. Onc U6shGFP and Onc miR-neg, which expresses a non-target pre-miRNA sequence under the control of the CMV promoter, were used as negative controls. These viruses were tested on the BHK-LacZ cell line, which constituently expresses β-galactosidase and is highly susceptible to HSV infection [32].

To investigate the properties of oncolytic HSV expressing shRNA or miRNA, we performed an MTS cell viability assay at 24, 48 and 72 hours on BHK-LacZ cells infected with the oncolytic backbone virus OncoVEX, Onc U6shLacZ or Onc miR-LacZ at MOIs 1.0, 0.1 and 0.01. Furthermore, we prepared growth curves for these viruses using the same time points. Figures 1g and 1h demonstrate that expression of shRNA/miRNA from the LAT region does not significantly influence the cytopathic effect and growth properties of oncolytic HSV, respectively. Figure 1g further demonstrates that cell death increases with time and MOI of virus.

BHK-LacZ cells infected with Onc U6shLacZ, Onc miR-LacZ or each of the negative controls (Onc U6shGFP and Onc miR-neg) at an MOI of 1.0, 0.1 and 0.01 were assayed for β-galactosidase expression at 24, 48 and 72 hours post-infection. At each MOI and time point tested, the number of cells and concentration of virus in each culture remain the same. Silencing of *lacZ *does not affect the cytopathic effect of the virus (Figure 1g) and thus, cell survival in cultures treated with controls, Onc U6shLacZ or Onc miR-LacZ is expected to be the same in each one of the conditions. To compare silencing between conditions with different levels of cell survival, β-galactosidase levels (% LacZ) were expressed as a percentage of the expression measured in the cells infected with the negative controls (Onc U6shGFP for Onc U6shLacZ, and Onc miR-neg for Onc miR-LacZ). Analysis of β-galactosidase expression using enzyme activity assay revealed that both viruses which targeted *lacZ *significantly reduced β-galactosidase expression levels compared to the negative control viruses at most MOIs and timepoints tested, with the virus expressing artificial miRNA being consistently more effective than the virus expressing shRNA (Figure 1c,d,e).

At the earliest time point tested (24 hours), the most effective silencing occurred at the highest MOI of 1.0 (Onc U6shLacZ: 52.2% silencing; Onc miR-LacZ: 72.1% silencing, P = 0.0012), whereas the least effective silencing was observed at the lowest MOI of 0.01 (Onc U6shLacZ: 1.3% silencing; Onc miR-LacZ: 11.0% silencing, P = 0.0033) (Figure 1c). At 48 hours post-infection, both viruses resulted in increased β-galactosidase knockdown at all MOIs tested compared to the negative controls. However, the most effective silencing occurred at the lower MOI of 0.1 (Onc U6shLacZ: 69.7% silencing; Onc miR-LacZ: 80.0% silencing, P = 0.003) (Figure 1d). At 72 hours post-infection, silencing at the high MOIs was further reduced and the most effective silencing was observed with Onc miR-LacZ at an MOI of 0.01 (80.0% silencing) (Figure 1e).

Figure 1f demonstrates β-galactosidase activity levels in cells infected with Onc U6shGFP and Onc miR-neg control viruses. All BHK-LacZ cells constituently express β-galactosidase and thus, β-gal activity levels are a good indication of the number of cells that have survived at each MOI and time point. As expected from figure 1g, β-galactosidase levels decrease with time and MOI of virus. At an MOI 1.0, silencing is optimal at 48 hrs, when half of the cells in the culture remain healthy, and minimal at 72 hrs, when the majority of cells are killed by the virus. At an MOI 0.01, however, optimal silencing is achieved at 72 hrs, because the majority of cells in the culture remain healthy. Since β-galactosidase levels in BHK-LacZ cells infected with negative controls appear to decrease with time and MOI of virus (Figure 1f) as a result of cell death induced by the oncolytic backbone (Figure 1g) and neither the cytotoxicity nor the growth of oncolytic HSV are significantly affected by expression of RNAi triggers (Figure 1g and 1h), a significant further reduction in the levels of β-galactosidase in cells infected with oncolytic HSV expressing either shLacZ or miR-LacZ (Figures c, d and e) can only be attributed to an RNAi-specific effect.

Collectively, the above results indicate that in order to achieve optimal levels of silencing from a replicating HSV, it is important to balance the levels of shRNA/miRNA expression against cytotoxicity caused by the virus. Insertion of the same shRNA and miRNA expression cassettes in the LAT region of replication-defective HSV viruses resulted in expression of RNAi triggers in BHK-LacZ cells in the complete absence of viral replication [32]. Although expression of RNAi triggers from the LAT region is not dependant on viral replication, the initial number of cells infected and number of viral particles per cell (i.e. MOI) will determine the rate at which RNAi triggers are expressed. As viral genome copies increase exponentially and cell growth rate decreases, even at very low MOIs, the virus will eventually infect all the cells in the culture and complete cytopathic effect (CPE) will be achieved. At high MOIs, high levels of RNAi triggers are expressed at early time points, but if the MOI is too high, the cells may not remain healthy long enough to allow the RNAi triggers to act. Conversely, at low MOIs, high levels of RNAi triggers will be expressed at later time points, but the cells remain healthier longer to allow the RNAi triggers to induce effective silencing.

Finally, expression of miRNA from the CMV polymerase II promoter silenced target gene expression more efficiently than expression of shRNA from the U6 polymerase III promoter at every time point and MOI tested (unpaired student t tests). These results contrast with data in BHK-LacZ cells using non-replicating HSV expressing shRNA or miRNA from the same cassettes, which showed no significant difference between shRNA expressed from different promoters [32]. On the basis of these results Onc miR-LacZ was used for the *in vitro *and *in vivo *tumour cell studies described below.

### Oncolytic HSV-mediated silencing in glioma cells in culture

Oncolytic HSV has been shown to infect a wide range of tumour cell types, including glioma [38-42], colon carcinoma [43], prostate cancer [44,45], non-small cell lung cancer [46], gallbladder carcinoma [47], head and neck squamous cell carcinoma [48], oesophageal adenocarcinoma [49], breast cancer [50], thyroid cancers [51], hepatocellular carcinoma [52] and neuroblastoma [53,54], with variable efficiencies. The HSV genome, therefore, potentially offers a multifunctional platform from which to knockdown tumour-related transcripts, which could be used to help limit tumour immune suppression or tumour growth as a whole.

The 9L/lacZ rat gliosarcoma cell line was developed from the parental 9L cell line by transduction with the BAG replication-deficient retroviral vector carrying the *E. coli *lacZ gene. These cells constitutively express high levels of β-galactosidase from the 9L genome and are moderately susceptible to HSV infection [30]. Moderately susceptible tumour cells allow HSV entry and replication, but at a much lower rate than that required for effective tumour killing. As a result, a lower number of cells in a tumour will get infected by the virus at any given dose than in more susceptible cell types. While the virus will eventually kill infected cells, the process is slower. The lower level of viral replication will also result in a lower number of infectious particles being released to infect neighbouring tumour cells. When the kinetics of tumour cell killing do not exceed tumour growth, expression of shRNA or miRNA in combination to the viral cytopathic effect may be beneficial. Expression of RNAi triggers from the LAT region is not dependant on viral replication and will therefore result in the production of numerous silencing molecules from a single viral genome copy, which has the potential to improve the oncolytic properties of HSV by compensating for low viral particle numbers. Furthermore, the oncolytic process is slower, which may allow silencing molecules to accumulate and induce effective silencing of targeted genes before infected cells are killed by the virus. We have previously shown *in vivo *that OncoVEX replication inhibits the growth of 9L tumours in the flanks of nude F344 rats but fails to cure the tumours [30]. Similar results were obtained with this virus in RG2 glioma cells. Furthermore, expression of prodrug activation genes from this oncolytic backbone enhanced the cytopathic effect of the virus in 9L cells [30]. 9L cells are therefore an appropriate model in which to test oncolytic HSV-mediated silencing.

**Figure 2:**
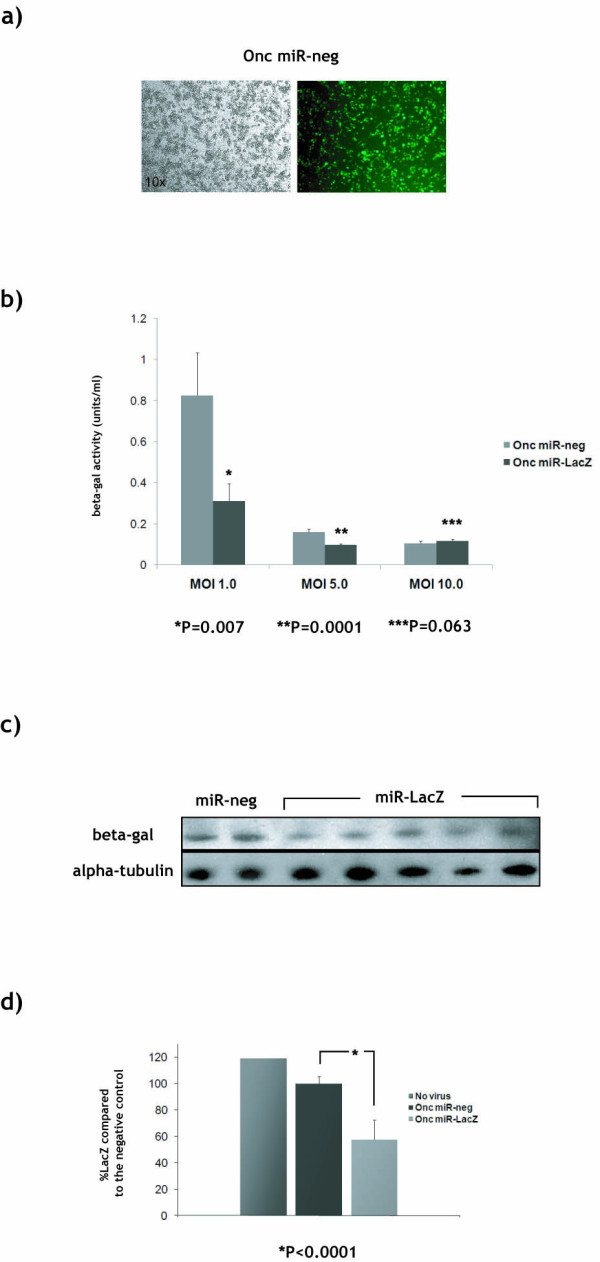
**Silencing in glioma cells *in vitro *and *in vivo***. **(a) **Rat 9L/LacZ [30] cells were infected at an MOI of 1.0 with Onc miR-neg and incubated for 48 hrs. GFP levels were assessed by fluorescent microscopy. **(b) **Rat 9L/LacZ cells were infected at an MOI of 1.0, 5.0 and 10.0 with Onc miR-LacZ or Onc miR-neg, and incubated for 48 hrs. B-galactosidase protein levels (units/ml) were assessed using high sensitivity β-galactosidase assay (Stratagene). P values were obtained using unpaired student t test. (**c,d**) Rat 9L/LacZ cells were implanted (1×10^7 ^cells) subcutaneously in the right flank of BALB/c nude mice (Harlan laboratories). Tumours were allowed to develop for 2 weeks to an average diameter of approximately 0.4-0.6 cm and then injected with 10^8 ^pfu (100 μl) of either Onc miR-LacZ or Onc miR-neg, and further incubated for 72 hrs. B-galactosidase protein levels were either assessed by standard (**c**) Western blot techniques or (**d**) high sensitivity β-galactosidase assay kit (Stratagene).

To test whether expression of miRNA from oncolytic HSV could achieve silencing in tumour cells in culture, we infected the 9L/LacZ cells with Onc miR-LacZ or Onc miR-neg, at an MOI of 1.0, 5.0 and 10.0. Optimal silencing in BHK cells was achieved at 48 hrs, MOI 0.1. 9L cells are moderately susceptible to HSV infection compared to BHK cells and thus, considerably higher MOIs were used. B-galactosidase activity assay at 48 hrs post-infection demonstrated 62.5% silencing at MOI 1.0 (P = 0.0007), 34.7% silencing at MOI 5.0 (P = 0.0001) and no silencing at MOI 10.0 (Figure 2b). Figure 2b further demonstrates that 5 times more cells were killed by the virus at MOI 5.0 compared to MOI 1.0, and 1.5 times more cells were killed at MOI 10.0 compared to MOI 5.0. To investigate the reason *lacZ *silencing achieved in these cells was moderate compared to silencing in BHK-LacZ cells, we assessed the levels of GFP expressed from miR viruses that enable direct labelling of infected cells. Fluorescent microscopy in 9L/LacZ infected with Onc miR-neg at 48 hrs revealed that only a proportion of cells are infected at an MOI 1.0 (Figure 2a). At MOIs 5.0 and 10.0, nearly all cells in the cultures are killed by the virus (data not shown). Thus, that optimal silencing in 9L cells at 48 hrs can be achieved between MOI 1.0 and 5.0, i.e. at considerably higher doses than those needed to achieve near complete silencing in a highly susceptible cell line.

The above data revealed that expression of miRNA from an oncolytic HSV virus can induce silencing in a tumour cell line. Although the 9L/LacZ rat glioma cell line is infected with relatively low efficiency and thus, *lacZ *is only partially silenced, this cell line is an appropriate model to study oncolytic HSV-mediated silencing, as silencing is expected to be particularly useful in tumours which are less efficiently infected by the virus and thus, not completely killed by oncolytic HSV treatment.

### Oncolytic HSV-mediated silencing in tumours *in vivo*

To investigate oncolytic HSV-mediated silencing in tumours *in vivo*, we injected the flanks of nude mice with 9L/LacZ cells and allowed tumours to develop. The resulting tumours were injected with 10^8 ^pfu of either Onc miR-LacZ or Onc miR-neg and were analysed for β-galactosidase expression by Western blot and enzyme activity assay 72 hrs post-injection. Quantitative RT-PCR revealed similar levels of GFP transcripts within tumours injected with either virus (data not shown). Western blot analysis of tumours isolated from animals injected with Onc miR-LacZ (n = 4) demonstrated knockdown of β-galactosidase levels compared to tumours injected with Onc miR-neg (n = 2) (Figure 2c). Moderate reduction in protein levels is difficult to convincingly demonstrate using western blot. Furthermore, β-galactosidase is highly expressed in 9L tumour cells and has a very long half-life resulting in high background, which makes quantification of western blots using band density very difficult. For these reasons, knockdown of β-galactosidase was confirmed using β-gal enzyme activity assay, which is quantitative and considerably more sensitive than western blot. B-galactosidase activity assay on the same tumours revealed that non-injected tumours expressed the highest levels of β-galactosidase (n = 2), injection with Onc miR-neg resulted in an average 19.4% silencing of lacZ (n = 2) compared to non-injected tumours (P = 0.005), and injection with Onc miR-LacZ resulted in 49 ± 5.9% silencing of lacZ (n = 4) compared to tumours injected with the Onc miR-neg control (P = 0.0001) (Figure 2d). We have previously shown that the oncolytic virus used in this study can inhibit the growth of 9L tumours in the flanks of F344 rats [30]. Therefore, we can conclude, that the 19.4% difference in the levels of β-galactosidase between non-injected tumours and tumours injected with miR-neg control is due to oncolysis caused by the viral backbone. 49% silencing in tumours *in vivo *is a highly promising result bearing in mind that 9L/LacZ cells are moderately susceptible to HSV infection. These results indicate that an oncolytic HSV expressing shRNA/miRNA has the potential to silence target gene expression in tumours *in vivo*, which had not been previously demonstrated.

## Discussion

Unlike oncolytic adenoviral vectors, which replicate at a very slow rate and have been shown to induce RNAi in tumours [18-20], replicating HSV was not considered a suitable vector from which to express RNAi triggers, because it is robustly oncolytic. This study is the first to show oncolytic HSV-mediated expression of shRNA and artificial miRNA. Our results demonstrate effective and specific silencing of a reporter gene in tumour cells both *in vitro *and *in vivo*. We also demonstrate that expression of shRNA from an oncolytic HSV backbone can be driven from either a polymerase II or III promoter, although expression from the CMV promoter was comprehensibly more effective. The use of artificial miRNAs has become a very attractive alternative to the expression of shRNA. Artificial miRNAs are amenable to RNA polymerase II transcription and polycistronic strategies, which allow delivery of multiple shRNA sequences simultaneously and co-expression of a biomarker or biologically active protein together with the shRNA.

Furthermore, we show that oncolytic HSV-mediated silencing can be achieved in cell types that are either highly susceptible or moderately susceptible to HSV infection. We show that when oncolytic HSV is given at the right dose, shRNA can be expressed and effective silencing can be induced in an infected cell, before the cell is killed by the virus. Thus, delivery of RNAi to tumours using oncolytic HSV provides the potential to improve tumour cell killing by silencing tumour-related genes. For example, induction of RNAi in combination with the virus oncolytic effect may be able to improve response rates seen in human clinical trials with oncolytic viruses. Moreover, expression of shRNA from oncolytic HSV may be useful in enhancing cell killing in tumours that are relatively less susceptible to HSV infection. We show that expression of RNAi triggers from the LAT region, which is not dependant on viral replication, is sufficient to induce silencing of a target gene despite a low viral particle number in tumours where HSV infection and/or replication are moderate. When the shRNA or miRNA is engineered to target proteins secreted by tumour cells, which aid their growth or promote local immune suppression, moderate silencing in these tumours may be sufficient to promote cell death in both infected and non-infected cells in a tumour.

As the process of lytic virus replication is effective at destroying injected tumours, the most appropriate targets for RNAi-mediated knockdown using this approach would be expected to be secreted tumour proteins involved in processes such as angiogenesis or local immune suppression. Vascular endothelial growth factor (VEGF) is a key regulator of angiogenesis. Up-regulation of VEGF is important in blood vessel formation in solid tumours [55] and mediates tumour evasion of immune surveillance by inhibiting the development of dendritic and other hematopoetic cells [56]. In recent years, numerous studies have demonstrated that silencing of VEGF by non-viral-mediated delivery of siRNA leads to reduction in tumour size of up to 90% [57-60]. To improve delivery of RNAi triggers against VEGF, non-replicating viruses were generated [61,62]. More recently, replication-competent adenoviruses expressing shRNA against VEGF and Interleukin-8 were shown to affect angiogenesis and inhibit tumour growth [19,20]. Other factors secreted by tumours, such as Interleukin-10 (IL-10) and transforming growth factor-β (TGF-β), are attractive targets for knockdown using oncolytic HSV. IL-10 is an anti-inflammatory, immunosuppressive cytokine that is involved in tumour escape from immune surveillance [63]. Elevated IL-10 levels have been found in a variety of human malignant tumours and are an independent prognosis factor for decreased response to chemotherapy in patients with advanced gastrointestinal malignancies [64,65]. In a malignant B-1 cell line derived from a murine model of chronic lymphocytic leukaemia, induction of RNAi against IL-10 resulted in anti-proliferative and pro-apoptotic effects [66]. TGF-β is a multifunctional polypeptide which switches its role from a tumour suppressor in normal cells to a tumour promoter in advanced cancers. TGF-β protein-receptor interactions promote processes such as immune suppression, tissue remodelling and formation of blood vessels, which lead to the growth and metastasis of cancer cells [67]. TGF-β is the prototypic member of a large superfamily of secreted proteins that include three TGF-β isoforms (TGF-β1, TGF-β2 and TGF-β3). Antisense oligonucleotides against TGF-β1 developed for the treatment of non-small cell lung carcinoma, colorectal and prostate carcinomas have shown efficacy in preclinical development [68]. Antisense oligonucleotides against TGFβ2 have been tested in a number of clinical trials against various cancers, with promising results [69].

Infection with wild-type HSV results in expression of virion host shutoff protein (vhs), which leads to mRNA degradation and shutdown of host cell protein synthesis. However, whilst a proportion of cellular mRNAs that mainly encode proteins involved in the cell immune response to virus infection are degraded, microarray analysis of gene expression has demonstrated that almost 500 genes are up-regulated more than 3-fold in HSV-1-infected cells, compared with mock-infected cells [70]. Moreover, whilst expression of GFP from a non-replicating HSV virus (Figure 1b) is not likely to be affected by vhs, due to viral mRNA being transcribed at a higher rate than cellular mRNA, we demonstrate that lacZ, whose expression in 9L/LacZ cells resembles that of an endogenous gene, is not affected by vhs and can be specifically silenced both *in vitro *and *in vivo *(described in figure 2). Thus, although expression of vhs may alleviate the need to target certain cellular genes, expression of shRNA from an oncolytic HSV has the potential to allow specific silencing of genes that are not downregulated by HSV infection itself.

## Conclusion

In summary, the current study provides the first demonstration that an oncolytic HSV can be used to induce RNAi to tumour cells *in vitro *and tumours *in vivo*. A combination of oncolytic HSV and RNAi technology offers the potential to further increase the effectiveness of oncolytic HSV therapy by inhibiting genes involved in tumour-related processes which may enhance the overall anti-tumour effect, especially in tumours that are only moderately susceptible to HSV replication. The vectors described in this study are being engineered to target a selection of tumour-related genes, and experiments are underway to investigate whether the effectiveness of oncolytic virus treatment can be improved in tumours moderately susceptible to HSV infection when these targets are silenced.

## Competing interests

Dr. Guy Simpson is a former employee of BioVex Inc. (a commercial biotech company) and has no shares or share options in the company. Dr. Simpson left the company in April 2007. Dr. Anna-Maria Anesti is a former employee of BioVex Inc. and has no shares or share options in the company. Dr. Anesti has been a BioVex consultant since December 2008. Dr. Toby Price is an employee of BioVex Inc. and has share options in the company. Prof. Hardev Pandha is a physician and academic scientist with no commercial link to BioVex Inc. Dr. Rob Coffin is the chief technical officer of BioVex Inc. with both shares and share options in the company. BioVex Inc has funded the work described in this manuscript.

## Authors' contributions

AMA and GS have contributed equally to this work. GS constructed the oncolytic viruses and performed all experiments described in figure 1. AMA constructed the RNAi cassettes and performed all experiments described in figure 2. TP produced a high titre preparation of the OncoVex miR-neg and OncoVex miR-LacZ viruses. Authors AMA, GS, TP, HP, and RC had full access to all the data in the study and take responsibility for the integrity of the data and the accuracy of the data analysis. *Study concept and design: *AMA, GS, and RC. *Acquisition of data: A*MA and GS. *Analysis and interpretation of data: A*MA, GS, and RC. *Drafting of the manuscript: *AMA, GS, HP and RC. *Statistical analysis: *GS. *Study supervision: *HP and RC. All authors read and approved the final manuscript.

## Pre-publication history

The pre-publication history for this paper can be accessed here:

http://www.biomedcentral.com/1471-2407/10/486/prepub
